# Orofacial pain and dysfunction in patients with Parkinson**'**s disease: A scoping review

**DOI:** 10.1002/ejp.2031

**Published:** 2022-09-16

**Authors:** Merel C. Verhoeff, Michail Koutris, Sharine Tambach, Denise Eikenboom, Ralph de Vries, Henk W. Berendse, Karin D. van Dijk, Frank Lobbezoo

**Affiliations:** ^1^ Department of Orofacial Pain and Dysfunction, Academic Centre for Dentistry Amsterdam (ACTA) University of Amsterdam and Vrije Universiteit Amsterdam Amsterdam The Netherlands; ^2^ Medical Library Vrije Universiteit Amsterdam The Netherlands; ^3^ Amsterdam University Medical Centres (Amsterdam UMC) Vrije Universiteit Amsterdam, Neurology, Amsterdam Neuroscience Amsterdam The Netherlands; ^4^ Sleep Wake Centre Stichting Epilepsie Instellingen Nederland (SEIN) Heemstede The Netherlands

## Abstract

**Background:**

Parkinson's disease (PD) is commonly known as a disorder that affects the smooth performance of body movements. In addition to the motor impairments, patients with PD often experience pain. Both motor impairments and pain can occur throughout the body, hence including the orofacial region. However, currently, there is a lack of knowledge on the orofacial manifestations. Since orofacial pain and dysfunction can, amongst others, reduce the quality of life of patients with PD, it is important to explore the prevalence of these symptoms in the PD population.

**Objective:**

To provide a broad overview of the relevant literature on orofacial pain and dysfunction in patients with PD. Furthermore, we aim to generate hypotheses for future research on this topic.

**Databases and data treatment:**

A literature search (in PubMed, Embase.com, Web of Science [Core collection], and Cochrane Library) was performed on 20 January 2022, in collaboration with a medical librarian. In total, 7180 articles were found, of which 50 were finally included in this scoping review.

**Results:**

In the included studies, pain (e.g. orofacial pain (*N* = 2) and temporomandibular disorder pain (*N* = 2)), orofacial motor dysfunction (e.g. limited jaw movements (*N* = 10), reduced maximum muscle output (*N* = 3), chewing difficulties (*N* = 9), unspecified TMD (*N* = 3), sensory disturbances (*N* = 1)), and bruxism (*N* = 3) were observed more often in patients with PD than in healthy controls.

**Conclusion:**

Patients with PD experience more pain in the orofacial area and more dysfunction of the masticatory system than their healthy peers.

**Significance:**

This scoping review can increase health care providers' awareness of the problems that can be encountered in the orofacial area of PD patients, especially pain syndromes also occur in the orofacial region and not only in the extremities. Besides, dysfunction of the orofacial area is elaborated in this scoping review, which helps to understand that this limits PD patients' quality of life. Further, the outcomes of this scoping review can assist in encouraging collaboration between medicine and dentistry. Finally, this scoping review suggests new research areas, based on the gaps identified in the current literature on this topic. Ultimately, this will improve individualized strategies for reducing orofacial pain and/or dysfunction in PD patients.

## 1 INTRODUCTION

Parkinson's Disease (PD) is a neurodegenerative disorder characterized by the accumulation of alpha‐synuclein in Lewy Bodies (Bloem et al., 2021) and neuronal loss in specific brain areas, amongst others in the substantia nigra (Kalia & Lang, 2015). In total, 1–4% of adults older than 60 years of age are affected by this disease (Eimers et al., 2019). PD's most familiar clinical appearance is associated with motor symptoms, such as rigidity, tremor, and bradykinesia. However, even though non‐motor features of PD are less familiar, they are also commonly present. Examples are depression, sleep disorders, cognitive dysfunction, and pain (Khoo et al., 2013). Pain in patients with PD has a prevalence ranging between 68% and 85% (Beiske et al., 2009). One of the most common types of pain in this patient group is musculoskeletal pain (Beiske et al., 2009).

Orofacial pain is defined as “a frequent form of pain perceived in the face and/or oral cavity”. It consists of different types of pain syndromes and/or disorders (International Classification of Orofacial Pain (ICOP), 2020). For example, temporomandibular disorders (TMD) is a collective term that embraces disorders of the temporomandibular joint, the masticatory muscles, and adjacent structures (de Leeuw & Klasser, 2018). Symptoms of TMD include orofacial pain and headaches attributed to TMD, as well as dysfunction of the masticatory system, including joint sounds and limitations in the movement of the mandible (Schiffman et al., 2014). Although not fully elucidated yet, the aetiology of TMD is considered multifactorial, with combinations of a host of biopsychosocial factors playing a role, amongst which bruxism (Lobbezoo & Naeije, 2001; Manfredini et al., 2021). Bruxism is defined as “a repetitive jaw‐muscle activity characterised by clenching or grinding of the teeth and/or by bracing or thrusting of the mandible” (Lobbezoo et al., 2018). In addition, bruxism encompasses two circadian forms, namely sleep bruxism and awake bruxism (Lobbezoo et al., 2018). Another disorder that may be accompanied by pain in the orofacial area is burning mouth disorder. It is defined as “an intraoral burning or dysaesthetic sensation, recurring daily for more than 2 hours per day over more than 3 months, without evident causative lesions on clinical examination and investigation” (International Classification of Orofacial Pain (ICOP), 2020).

According to Mylius et al. (2021), pain in PD patients could be part of the disease itself or could be unrelated to PD. So far, knowledge of orofacial pain and dysfunction in patients with PD is limited. In a previous study that assessed self‐reported complaints of orofacial pain and dysfunction in PD patients, a higher prevalence of TMD pain and sleep and awake bruxism was observed in this population (Verhoeff et al., 2018). In addition, lower velocity and deviated patterns of jaw movements have been observed in experimental animal studies using a primate model of PD (Adachi et al., 2012) as well as in humans diagnosed with PD (Albuquerque & da Silva, 2016). Furthermore, problems with mastication have been suggested to occur in association with these symptoms (Friedlander et al., 2009). In the same way as oral health problems, orofacial pain and dysfunction could negatively influence Oral Health‐Related Quality of Life (OHRQoL) in PD patients (Verhoeff et al., 2022). Although oral health problems in PD patients have not been studied extensively, they received more attention than orofacial pain and dysfunction (Verhoeff et al., n.d.; Auffret et al., 2021; Van Stiphout et al., 2018). We believe that more insight into both topics is essential, as to ultimately prevent orofacial problems in their broadest sense in PD patients.

Against this background, our scoping review aimed to give a broad overview of the relevant literature on the prevalence of orofacial pain and/or dysfunction in patients with PD and, whenever possible, in comparison with controls. Furthermore, we aimed to see which patient‐related characteristics are associated with orofacial pain and/or dysfunction in PD patients. Finally, we aimed to generate hypotheses for future research on this topic.

## 2 MATERIAL AND METHOD

### 2.1 Search strategy

A literature search was performed based on the Preferred Reporting Items for Systematic Reviews and Meta‐Analyses (PRISMA)‐statement (www.prisma‐statement.org) (Moher et al., 2009). To identify all relevant publications, we conducted systematic searches in the bibliographic databases PubMed, Embase.com, Clarivate Analytics/Web of Science (Core collection), and Wiley/Cochrane Library from inception to 20 January 2022, in collaboration with a medical librarian. The following terms were used (including synonyms and closely related words) as index terms or free‐text words: ‘Parkinsonian Disorders’, ‘Oral health’, ‘Oral functioning’ and ‘Quality of Life’(Verhoeff et al., n.d.). The reference lists of the identified articles were searched for relevant publications. Duplicate articles were excluded. The complete search strategies for all databases can be found in Table S1 (Verhoeff et al., n.d.).

### 2.2 Inclusion and exclusion criteria

Studies were included if they met the following a priori formulated criteria: (i) inclusion of patients with a diagnosis of PD; (ii) information on oral health‐related factors (viz., orofacial pain, TMD‐pain, burning mouth disorder, jaw movements, maximum mouth output, chewing difficulties, unspecified TMD, non‐painful TMD, sensory disturbances, and bruxism); (iii) written in English or Dutch language. In addition, we excluded studies based on the following criteria: (i) the full text could not be retrieved, or it was not available; (ii) information on oral health‐related factors other than orofacial pain or dysfunction (e.g. caries, periodontitis and dental status; Verhoeff et al., n.d.); (iii) publication types that did not yield original data (e.g. editorials and [systematic] reviews of the literature).

### 2.3 Study selection

Three reviewers (MV, DE, and ST) independently screened all potentially relevant titles and abstracts for eligibility. Upon completion of the screening of all titles and abstracts, two reviewers (DE and ST) independently screened the full‐text articles of the included abstracts. Disagreements or doubts were dissolved through a consensus procedure with the third reviewer (MV).

### 2.4 Data extraction and analysis

All included studies were analysed using descriptive statistics, mainly percentages and means, including the standard deviations. In addition, all results were divided into: (i) within‐group results, to analyse the results for all PD patients; (ii) between‐group results, when a control group was included and comparisons could therefore be made. As part of this procedure, the following assumptions and choices were made (International Classification of Orofacial Pain (ICOP), 2020): (i) when it was not explicitly reported, but we could reasonably assume that TMD pain was reported (e.g. pain in jaw muscles, pain related to masticatory function) and not orofacial pain as an umbrella term, data was recorded as such; (ii) when articles did not make a clear distinction between TMD pain or dysfunction, articles were reported as ‘unspecified TMD’; (iii) when a distinction was made between ON‐ and OFF‐periods (i.e. dopaminergic therapy is either working [ON] or is not working or works suboptimal [OFF]), we chose to report the prevalence in OFF‐periods unless otherwise described; (iv) when chewing ability was measured with food particles or parafilm, we chose to report the objectified measurement of parafilm, unless described otherwise; (v) the maximum mouth opening was measured from the incisal edge of the maxillary central incisor to the incisal edge of the mandibular central incisor, unless reported otherwise; (vi) when a distinction was made between right and left, the right side was reported.

## 3 RESULTS

### 3.1 Search results

**FIGURE 1:**
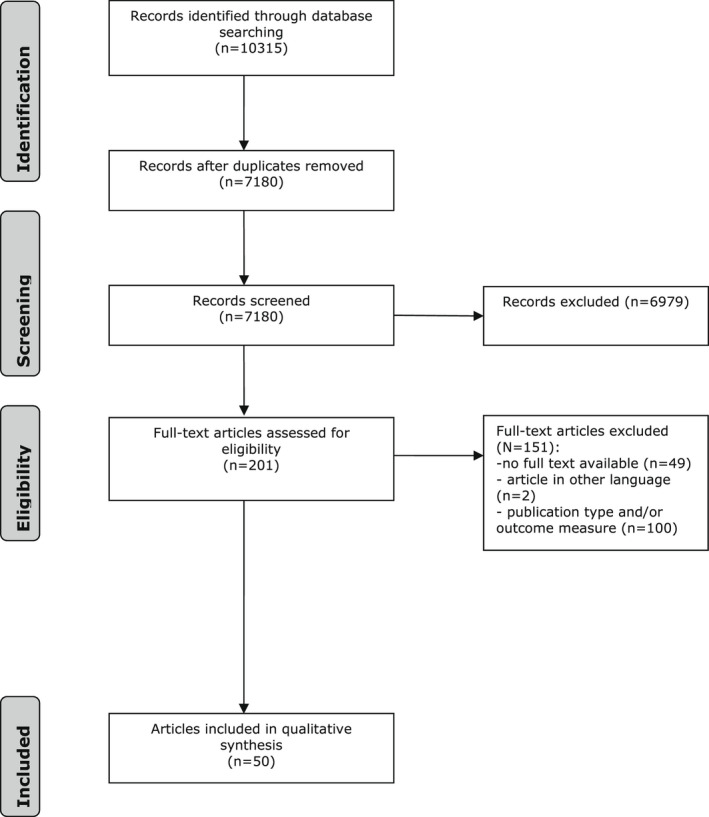
Flow chart of the search and selection procedure

In total, 10,315 articles were found: 497 in Cochrane Library, 4695 in embase.com, 2860 in PubMed, and 2263 in Web of Science. After removing duplicates, 7180 references remained. Following the study‐selection procedure, 50 studies performed in 18 different countries and published between 1970 and 2022 were included: 5 RCTs, 27 case‐control studies, 14 cross‐sectional studies, and 4 case reports. The flow chart of the search and selection procedure is presented in Figure 1. In addition, the characteristics of all included studies and their participants are shown in Table 1.

**TABLE 1:** Characteristics and demographics of the included studies (*N* = 40) and participants (*N* = 11.626)

Study	Country	Design	Np [N]	Nc [N]	Age PD M ± SD [range]	Male gender PD [N (%)]	Duration disease M ± SD Or [range]	Disease severity MDS‐UPDRS (part III) M ± SD or [range]	Disease severity HY scale M ± SD or [range]	APM [Y/N]	LEDD M ± SD mg/day or [range]	Outcome
Abe et al., 2013	Canada	CC	15	9	67.1 ± 2.6	12 (80.0%)	5.0 ±?	25.0 ± 2.9	3 ±?	Y	?	BR
Adachi et al., 2012	Japan	CC	3	3	N/A	3 (50.0%)	[0.5–0.8]	?	?	?	?	JM; CD
Adams et al., 2004	Canada	CC	10	10	?	?	?	?	?	Y	?	JM
Adewusi et al., 2018	UK	CC	51	51	68.3 ± 8.4	37 (72.5%)	11.4 ± 6.1	?	2.0 ±?	Y	?	OP
Agrawal et al., 2021	India	CS	100	N/A	62 [38–85]	75 (75.0%)	4.9 ± 4 [0–20]	15.0 ± 7.6	?	Y	414.1 ± 319.0	OP
Albuquerque & da Silva, 2016	Brazil	CC	2	1	?	?	?	?	?	?	?	JM
Anastassiadou et al., 2002	Greece	CS	51	N/A	67.5 ± 2.8	32 (63.0%)	10.1 ± 5.4	?	2.6 ± 0.9	?	?	CD
Bakke et al., 2011	Denmark	CC	15	15	[61–82]	6 (40.0%)	6.7 ± 3.8	[17–61]	[2–4]	Y	?	TMD pain; JM; CD; U‐TMD; SD
Bandini et al., 2016	Italy	CC	14	14	71.6 ± 7.0	9 (64.3%)	8.4 ± 6.1	16.0 ± 12.0	2.0 ± 0.3	Y	?	JM
Baram et al., 2020	Denmark	RCT	29	N/A	65.0 ± 10.0	15 (51.7%)	11.7 ± 5.0	20.6 ± 4.9	2.9 ± 0.4	Y	?	JM; CD; U‐TMD
Baram et al., 2021	Denmark	RCT	29	N/A	65 [32–79]	15 (51.7%)	11.7 ± 5.0	20.6 ± 4.9	2.9 ± 0.4	Y	?	CD; U‐TMD
Tavares et al., 2021	Brazil	CS	15	N/A	69.0 ±?	10 (66.7%)	?	?	[1–3]	?	?	U‐TMD
Baumann et al., 2020	Hungary	CC	35	42	62.9 ± 9.8	?	?	?	?	?	?	U‐TMD
Behari et al., 2020	India	CS	119	N/A	64.3 ± 9.6	83 (69.7%)	7.7 ± 5.6	15.9 ± 13.8	2.3 ± 0.9	Y	?	OP; TMD pain; BM
Bonenfant et al., 2016	Canada/France	CS	198	N/A	69.0 ± 10.3	116 (57.1%)	[6–10]	?	2.5 ±?	Y	630.1 ±?	OP; TMD pain; BM; BR
Choi et al., 2021	Korea	CH	6482	508,383	?	2858 (47%)	?	?	?	?	?	U‐TMD
Coon & Laughlin, 2012	USA	CR	1	—	65.0	1 (100%)	?	?	?	Y	[75–450]	BMD
Clifford & Finnerty, 1995	Ireland	CS	228	—	69.5^#^	121 (53%)	?	?	?	?	?	BMD
Clifford et al., 1998	Ireland	CS	115	—	70.0 ±?	65 (57%)	?	?	?	Y	?	BMD
da Silva et al., 2019	Brazil	CC	12	12	66.1 ± 3.3	?	?	?	[1–3]	Y	?	MMO
de Mattos et al., 2019	Brazil	CS	54	N/A	66.0 ±?	38 (70.4%)	4.0 ±?	?	?	y	?	OP; TMD pain; BM
Donizetti Verri et al., 2019	Brazil	CC	12	12	66.1 ± 3.3	?	?	?	[1–3]	Y	?	JM; MMO
Ford et al., 1996	USA	CR	7	N/A	70.1 ± 9.7	1 (14.3%)	?	?	?	Y	?	BMD
Garcia et al., 2021	Spain	CH	50	N/A	68.5 ± 9.1	21 (42.0%)	6.4 ± 5.1	?	?	Y	810.2 ± 518.1	OP
Gopalakrishnan et al., 2021	India	CS	50	N/A	[30–60]	41 (82%)	?	?	?	?	?	BMD
Karlsson et al., 1992	Sweden	CS	12	N/A	65.0 ±?	7 (58.3%)	8.0 ±?	?	[2–4]	Y	[50–300]	JM; CD
Katsikitis & Pilowsky, 1996	Australia	RCT	16	8	69.9 ± 5.8	7 (43.8%)	13.5 ± 12.2	?	?	Y	?	JM
Kwak et al., 2009	Korea	CS	45	N/A	73.0 ±?	18 (40.0%)	6.4 ±?	?	?	?	?	BR (AB)
Magee, 1970	?	CR	1	N/A	53	0	5	?	?	Y	?	BR
Martinez‐Martin et al., 2017	UK	CC	178	83	64.4 ± 11.4	38 (68.5%)	5.4 ± 4.9	?	2.72 ± 0.84	Y	?	OP; TMD pain; BM
Massimo et al., 2020	Italy	CS	24	24	71.4 ± 5.9	15 (62.5%)	?	9.4 ± 4.4	?	?	?	CD; U‐TMD
Minagi et al., 1998	Japan	CR	1	N/A	71	0	3.0	?	?	Y	?	TMD pain
Mylius et al., 2021	Switzerland & Brazil	CC	159	37	65.1 ± 11.6	99 (62%)	10.2 ± 7.6	35.5 ± 5.2	?	Y	1050 ± 635	TMD pain
Nakamura et al., 2013	Japan	CC	5	5	?	?	?	?	?	?	?	JM
Nakayama et al., 2004	Japan	CC	104	191	?	44 (42.3%)	?	?	?	?	?	CD
O'Neill et al., 2021	UK	CH	1916	N/A	68.0 ± 9.5	1272 (65.0%)	3.0 ± 2.1	?	?	Y	[400.0^−^465.0]	OP; TMD pain; BM
Persson et al., 1992	Sweden	CC	30	585	73.0 ± 7.3	17 (57.0%)	11.0 ± 5.4	?	?	Y	?	TMD pain; JM; CD; NP TMD; BR
Ribeiro et al., 2017a	Brazil	CS	17	17	69.4 ± 4.7	9 (52.9%)	6.8 ± 3.8	?	?	Y	?	JM; MMO; CD
Ribeiro et al., 2017b	Brazil	CS	17	17	69.4 ± 4.7	9 (52.9%)	6.8 ± 3.8	?	?	Y	?	CD
Robertson et al., 2011	USA	RCT	27	27	?	25 (92.6%)	?	?	?	Y	?	JM
Robertson et al., 2001	USA	RCT	6	10	51.5 ± 12.0	4 (66.6%)	?	?	3.9 ±?	Y	?	JM
Robertson & Hammerstad, 1996	USA	CC	8	11	53.7 ±?	6 (75.0%)	9.0 ±?	?	[2.5–4]	Y	?	JM
Rodríguez‐Violante et al., 2017	Mexico	CS	341	N/A	64.9 ± 12.0	182 (53.4%)	7.8 ± 5.0	29.7 ± 17.3	[1–5]	Y	674.0 ± 461.6	OP
Rodrigues Ribeiro et al., 2019	Brazil	CC	11	11	73.0 ± 3.2	6 (54.6%)	9.8 ± 3.8	?	?	Y	?	JM; MMO; CD
Da Costa Silva et al., 2015	Brazil	CS	59	N/A	65.4 ± 8.8	30 (50.8%)	7.1 ± 4.1	?	[1–3]	Y	?	U‐TMD
Silva et al., 2016	Brazil	CS	42	N/A	61.8 ± 1.8	21 (50.0%)	8.7 ± 4.6	?	[1–3]	?	?	U‐TMD
van Stiphout et al., 2018	Netherlands	CC	74	74	70.2 ± 8.8	48 (65.0%)	9.1 ± 6.4	?	2.4 ± 1.8	?	?	BM; CD
Verhoeff et al., 2018	Netherlands	CC	395	340	67.9 ± 8.6	232 (58.7%)	6.7 ± 5.9	?	?	Y	710.8 ± 469.8	TMD pain; NP TMD; BR
Verhoeff et al., 2022	Netherlands	CC	341	411	65.5 ± 8.4	60 (17.6)	7.0 ± 5.5	?	?	?	?	TMD pain; BMD
Wooten Watts et al., 1999	USA	CC	100	100	67.7 ± 9.4	?	?	?	?	?	?	TMD pain; JM; CD; NP TMD; BR

Abbreviations: %, percentage; ?, unknown; APM, Anti Parkinsonian Medication; CC, case control; CH, cohort study; CR, case report/series; CS, cross‐sectional; H&Y, Hoehn & Yahr Scale; LEDD, Levodopa Equivalent Daily Dosages; M, Mean; MDS‐UPDRS, Movement Disorders Society Unified Parkinson Disease Rating Scale; mg/day, milligrams per day; N/A, Not Applicable; RCT, randomized controlled trial; SD, standard deviation; UK, United Kingdom; USA, United States of America.

### 3.2 Orofacial pain

#### 3.2.1 Orofacial pain

In total, nine studies reported unspecified pain in the orofacial area (Adewusi et al., 2018; Agrawal et al., 2021; Behari et al., 2020; Bonenfant et al., 2016; de Mattos et al., 2019; García et al., 2021; Martinez‐Martin et al., 2017; O'Neill et al., 2021; Rodríguez‐Violante et al., 2017), of which two included a control group (Adewusi et al., 2018; Martinez‐Martin et al., 2017). The within‐group results showed prevalences that varied between 12 and 74%. Furthermore, the between‐group results of the two studies that included a control group showed significantly higher orofacial pain scores in PD patients than the controls, indicating that PD patients experience more orofacial pain than their healthy peers (Table 2; Adewusi et al., 2018; Martinez‐Martin et al., 2017). Both studies used the King Parkinson's Pain Scale (KPPS), an inter‐rater‐based interview scale.

**TABLE 2:** Results for orofacial pain (viz., orofacial pain, TMD‐pain, and burning mouth disorder) in patients with PD compared to controls

Article	Method	Outcome measure		PD	Control	*p*‐value
Orofacial pain						
Adewusi et al., 2018	RIBS (KPPS)	OFPS	M ± SD	0.6 ± 2.0	0.0 ± 0.0	*p* ≤ 0.05
Agrawal et al., 2021	RIBS (KPPS)	OP	Prevalence	12%	N/A	N/A
Behari et al., 2020	RIBS (KPPS)	OP	Prevalence	14.8%	N/A	N/A
Bonenfant et al., 2016	SR (Quest)	OP	Prevalence	74.2%	N/A	N/A
García et al., 2021	RIBS (KPPS)	OFPS	M ± SD	2.5 ± 9.6	N/A	N/A
Martinez‐Martin et al., 2017	RIBS (KPPS)	OP	Prevalence	20.8%	?	?
	RIBS (KPPS)	OFPS	M ± SD	1.0 ± 3.0	0.2 ± 1.4	*p* ≤ 0.05
de Mattos et al., 2019	RIBS (KPPS)	OP	Prevalence	7.8%	N/A	N/A
O'Neill et al., 2021	RIBS (KPPS)	OP	Prevalence	7.3%	N/A	N/A
Rodríguez‐Violante et al., 2017	RIBS (KPPS)	OFPS	M ± SD	1.1 ± 3.6	N/A	N/A
	RIBS (KPPS)	OP	Prevalence	17.3%	N/A	N/A
TMD pain						
Bakke et al., 2011	CA (palpation)	Myalgia	M ± SD	0.2 ± 0.4	0.1 ± 0.3	*p* = 0.3
Bonenfant et al., 2016	SR (Quest)	Myalgia	Prevalence	3.4%	N/A	N/A
	SR (Quest)	Artralgia	Prevalence	9.5%	N/A	N/A
Behari et al., 2020	RIBS (KPPS)	OP‐chewing	Prevalence	6.5%	N/A	N/A
	RIBS (KPPS)	OP‐grinding	Prevalence	3.2%	N/A	N/A
Martinez‐Martin et al., 2017	RIBS (KPPS)	OP‐chewing	Prevalence	8.4%	1.2%	*p* ≤ 0.05
	RIBS (KPPS)	OP‐grinding	Prevalence	7.3%	2.4%	*p* = 0.1
de Mattos et al., 2019	RIBS (KPPS)	OP‐chewing	Prevalence	5.2%	N/A	N/A
	RIBS (KPPS)	OP‐grinding	Prevalence	5.2%	N/A	N/A
Mylius et al., 2021	RIBS (PCS)	Myalgia	Prevalence	25.0%	N/A	N/A
O'Neill et al., 2021	RIBS (KPPS)	OP‐chewing	Prevalence	2%	N/A	N/A
	RIBS (KPPS)	OP‐grinding	Prevalence	4%	N/A	N/A
Persson et al., 1992	SR (Quest)	OP‐chewing	Prevalence	0%	0.7%	NS
	SR (Quest)	Myalgia	Prevalence	33.3%	0.5%	NS
	CA (palpation)	Myalgia	Prevalence	10%^a^	35%^a^	*p* ≤ 0.01
	SR (Quest)	Artralgia	Prevalence	0%^a^	10%^a^	NS
	SR (Quest)	OP‐movement	Prevalence	6%^a^	5%^a^	NS
Verhoeff et al., 2018	SR (DC/TMD‐PS)	TMD pain	Prevalence	29.5%	19.1%	*p* ≤ 0.01
Verhoeff et al., 2022	SR (DC/TMD‐PS)	TMD pain	Prevalence	14.4%	N/A	N/A
Wooten Watts et al., 1999	SR (Quest)	Myalgia	Prevalence	13%	18%	NS
	SR (Quest)	TMD pain	Prevalence	3%	4%	NS
Burning mouth disorder						
Behari et al., 2020	RIBS (KPPS)	BM	Prevalence	4.8%	N/A	N/A
	RIBS (KPPS)	BM	M ± SD	0.21 ± 1.08	N/A	N/A
Bonenfant et al., 2016	SR (Quest)	BM	Prevalence	4.0%	N/A	N/A
Clifford & Finnerty, 1995	SR (Quest)	BM	Prevalence	9.7%	N/A	N/A
Clifford et al., 1998	SR (Quest)	BM	Prevalence	24%	N/A	N/A
Gopalakrishnan et al., 2021	SR + CA (?)	BM	Prevalence	30%	N/A	N/A
Martinez‐Martin et al., 2017	RIBS (KPPS)	BM	Prevalence	5.1%	1.2%	*p* = 0.13
de Mattos et al., 2019	RIBS (KPPS)	BM	Prevalence	2.6%	N/A	N/A
O'Neill et al., 2021	RIBS (KPPS)	BM	Prevalence	1.7%	N/A	N/A
van Stiphout et al., 2018	SR (Quest)	BM	Prevalence	4.1%	0%	*p* = 0.09
Verhoeff et al., 2022	SR (Quest)	BM	Prevalence	2.9%	N/A	N/A

Abbreviations: %, percentage; ?, not described; ?, unknown; BM, Burning Mouth; CA, Clinical Assessment; DC/TMD‐PS, Diagnostic Criteria for Temporomandibular Disorders – Pain Screener; KPPS, King's Parkinson's Pain Scale; M, Mean; N/A, Not Applicable; N/A, Not Applicable; NS, Not Significant; OFPS, Orofacial Pain Score; OP, Orofacial Pain; OP‐chewing, Orofacial Pain during chewing; OP‐grinding, Orofacial Pain during grinding; OP‐movement, Orofacial Pain during movement; OP‐movement, Orofacial pain during movement; *p*, p‐value; PCS, Parkinson's Disease Pain Classification System Questionnaire; Quest, Questionnaire; RIBS, Rater Interview Based Scale; SD, standard deviation; SR, Self‐report; TMD, Temporomandibular disorders.

^a^ Estimation because of reading figure.

In conclusion, orofacial pain in PD patients is more prevalent than in controls.

#### 3.2.2 Temporomandibular disorder pain (TMD pain)

Of the 11 studies that examined TMD pain (Bakke et al., 2011; Behari et al., 2020; Bonenfant et al., 2016; de Mattos et al., 2019; Martinez‐Martin et al., 2017; Mylius et al., 2021; O'Neill et al., 2021; Persson et al., 1992; Verhoeff et al., 2018; Verhoeff et al., 2022; Wooten Watts et al., 1999), five studies included a control group (Bakke et al., 2011; Martinez‐Martin et al., 2017; Persson et al., 1992; Verhoeff et al., 2018; Wooten Watts et al., 1999). The within‐group results showed prevalences that varied between 0 and 33%. When analysing the between‐group results, three of these studies showed significant differences between PD patients and controls (Table 2; Martinez‐Martin et al., 2017; Persson et al., 1992; Verhoeff et al., 2018). Martinez‐Martin et al. (2017) and Verhoeff et al. (2018) showed significantly higher TMD‐pain prevalence in PD patients than in controls. In contrast, the study of Persson et al. (1992) found a higher prevalence of myalgia (viz., during palpation) in controls than in PD patients. However, they did find a higher prevalence of fatigue in the masticatory muscles in PD patients (33%) than in controls (0.5%; Persson et al., 1992). Moreover, Bakke et al. (2011) and Wooten Watts et al. (1999) found more or less the same results for both groups. Only Bakke et al. (2011) and Persson et al. (1992) used clinical assessments to diagnose TMD pain. The other studies used the KPPS or a questionnaire. Finally, a case study described a PD patient who developed TMD pain located in her left joint, after involuntary movements involving that side of her face. After making a splint that restricted her movement, the pain disappeared (Table 4; Minagi et al., 1998).

In conclusion, TMD pain is suggested to be more prevalent in PD patients than in controls.

#### 3.2.3 Burning mouth disorder (BMD)

Of the 10 studies that examined BMD symptoms in PD patients (Behari et al., 2020; Bonenfant et al., 2016; Clifford & Finnerty, 1995; Clifford et al., 1998; de Mattos et al., 2019; Gopalakrishnan et al., 2021; Martinez‐Martin et al., 2017; O'Neill et al., 2021; van Stiphout et al., 2018; Verhoeff et al., 2022), two studies included a control group (Martinez‐Martin et al., 2017; van Stiphout et al., 2018). The within‐group results showed prevalences that varied between 1.7 and 30%. In addition, the between‐group results showed no significantly higher prevalence of BMD symptoms in PD patients than in the control group (Table 2). However, all studies except for Gopalakrishnan et al. (2021) used self‐reports to diagnose BMD. Gopalakrishnan et al. (2021) described that they used self‐reports and a clinical assessment; however, what kind of clinical assessment was used was not reported. Finally, a case study and a case series described six patients with PD, who developed a burning sensation in the oral cavity, gums, and face (Table 4; Coon & Laughlin, 2012; Ford et al., 1996).

In conclusion, the results suggest that the prevalence of BMD in a population of PD patients does not differ from that in controls.

### 3.3 Orofacial dysfunction

#### 3.3.1 Limited jaw movements

In total, 16 studies analysed limitations in the jaw movements of PD patients (Adachi et al., 2012; Adams et al., 2004; Albuquerque & da Silva, 2016; Bakke et al., 2011; Bandini et al., 2016; Baram et al., 2020; Donizetti Verri et al., 2019; Karlsson et al., 1992; Katsikitis & Pilowsky, 1996; Nakamura et al., 2013; Persson et al., 1992; Ribeiro et al., 2017a, 2017b; Robertson et al., 2001; Robertson & Hammerstad, 1996; Wooten Watts et al., 1999). Out of these, twelve studies included a control group (Table 3; Adachi et al., 2012; Adams et al., 2004; Albuquerque & da Silva, 2016; Bakke et al., 2011; Bandini et al., 2016; Donizetti Verri et al., 2019; Nakamura et al., 2013; Persson et al., 1992; Robertson et al., 2001; Robertson & Hammerstad, 1996; Wooten Watts et al., 1999). A distinction could be made between studies focussing on the amplitude of jaw movements, the self‐reported difficulties PD patients experienced during jaw movements or the velocity of the movements.

**TABLE 3:** Results for orofacial dysfunction (viz., limited jaw movements, maximum muscle output, chewing difficulties, unspecified TMD, non‐painful TMD, sensory disturbances, and bruxism) in patients with PD compared to controls

Orofacial dysfunction						
Article	Method	Outcome measure		PD	Control	*p*‐value
Limited jaw movements						
Adams et al., 2004	CA (habitual)	Duration of jaw movement (cycles/s)	M	2.5	3	*p* ≤ 0.05
Adachi et al., 2012	CA (exp)	Movement during opening (mm)	M	18^a^	24^a^	*p* ≤ 0.01
	CA (exp)	Movement during closing (mm)	M	11^a^	20^a^	*p* ≤ 0.01
	CA (exp)	Velocity of opening (mm/s)	M	125^a^	200^a^	*p* ≤ 0.01
	CA (exp)	Velocity of closing (mm/s)	M	150^a^	200^a^	*p* ≤ 0.01
Albuquerque & da Silva, 2016	CA (exp)	MMO (mm)	M	17.5^b^	36 mm	N/A
	CA (exp)	MMO (mm)	M	38.5^c^	36 mm	N/A
	CA (exp)	Velocity of MMO (mm/s)	M	213^b^	468	N/A
	CA (exp)	Deviation of opening path (mm)	M	9.7^c^	2.7 mm	N/A
Bakke et al., 2011	CA (exp)	MMO (mm)	M ± SD	44.0 ± 7.1	58.5 ± 4.3	*p* ≤ 0.01
Bandini et al., 2016	CA (exp)	Velocity of opening (mm/s)	M ± SD	94.94 ± 33.40	64.45 ± 30.94	*p* ≤ 0.05
	CA (exp)	Velocity of closing (mm/s)	M ± SD	87.85 ± 31.28	61.54 ± 28.49	*p* ≤ 0.05
	CA (exp)	Normalized range of opening	M ± SD	0.46 ± 0.23	0.65 ± 0.36	*p* = 0.10
Baram et al., 2020	CA (exp)	MMO (mm)	M ± SD	49.2 ± 6.9	N/A	N/A
Donizetti Verri et al., 2019	CA (rest)	RMS rest (CC sEMG)	MM	0.23	0.07	*p* ≤ 0.01
	CA (exp)	RMS maximum lateral movement (CC sEMG)	MM	0.33	0.12	*p* ≤ 0.01
	CA (exp)	RMS maximum protrusion (CC sEMG)	MM	0.47	0.14	*p* ≤ 0.01
Karlsson et al., 1992	CA (exp)	Velocity of closing (mm/s)	M ± SD	136 ± 30	N/A	N/A
	CA (exp)	Velocity of opening (mm/s)	M ± SD	188 ± 36	N/A	N/A
	CA (exp)	Duration of total movement cycle (s)	M ± SD	0.50 ± 0.13	N/A	N/A
Katsikis & Pilowsky, 1996	CA (habitual)	MO^d^ (mm)	M ± SD	15.3 ± 5.7	N/A	N/A
Nakamura et al., 2013	CA (exp)	Duty time (%) m. masseter (for activities 5% EMG‐peak)	M ± SD	6.0 ± 3.0^a^	5.0 ± 1.0^a^	NS
	CA (exp)	Duty time (%) m. digastricus (for activities 5% EMG‐peak)	M ± SD	18.2 ± 2.9%	13.1 ± 4.7%	*p* ≤ 0.05
Persson et al., 1992	CA (exp)	Opening difficulties (<40 mm)	Prevalence	25%	12.5%	*p* ≤ 0.05
	SR (Quest)	Opening difficulties	Prevalence	1%	0.7%	NS
Ribeiro et al., 2017a	CA (exp)	MMO (mm)	M ± SD	21.9 ± 12.7	34.8 ± 8.6	*p* ≤ 0.01
	CA (exp)	Lateral deviation (mm)	M ± SD	2.8 ± 2.9	6.7 ± 4.0	*p* ≤ 0.01
	CA (exp)	Maximum protrusion (mm)	M ± SD	18.9 ± 13.4	31.7 ± 8.4	*p* ≤ 0.01
	CA (exp)	Maximum lateral right (mm)	M ± SD	4.2 ± 3.0	12.6 ± 6.4	*p* ≤ 0.01
RodriguesRibeiro et al., 2019	CA (exp)	MMO (mm)	M ± SD	31.3 ± 3.1	N/A	N/A
	CA (exp)	Lateral deviation (mm)	M ± SD	2.2 ± 2.2	N/A	N/A
	CA (exp)	Maximum protrusion (mm)	M ± SD	41.6 ± 9.9	N/A	N/A
	CA (exp)	Maximum lateral right (mm)	M ± SD	6.7 ± 2.9	N/A	N/A
Robertson & Hammerstad, 1996	CA (exp)	MMO (mm)	M ± SD	28 ± 5^a^	40 ± 5^a^	*p* ≤ 0.01
	CA (habitual)	Velocity of opening (mm/s)	M ± SD	120 ± 50^a^	210 ± 75^a^	*p* ≤ 0.05
	CA (habitual)	Velocity of closing (mm/s)	M ± SD	120 ± 50^a^	210 ± 75^a^	*p* ≤ 0.05
	CA (exp)	MO (mm)	M ± SD	11 ± 4^a^	13 ± 4^a^	NS
Robertson et al., 2001	CA (exp)	MMO (mm)	Range	17.5–42	N/A	N/A
	CA (exp)	Velocity of opening (mm/s)	Range	25–170	N/A	N/A
	CA (exp)	Velocity of opening (mm/s)	M ± SD	105 ± 10^a^	180 ± 8.3	N/A
Wooten Watts et al., 1999	SR (Quest)	Opening difficulties	Prevalence	4%	4%	NS
Maximum muscle output						
Donizetti Verri et al., 2019	CA	Bite force (N)	M ± SD	164.6 ± 96.76	400.5 ± 224.50	*p* ≤ 0.01
	CA (rest)	Muscle thickness, masseter (cm)	M ± SD	0.78 ± 0.20	1.00 ± 0.16	*p* ≤ 0.05
	CA (rest)	Muscle thickness, temporalis (cm)	M ± SD	0.74 ± 0.15	0.59 ± 0.16	*p* ≤ 0.05
	CA (MVC, electrical)	Muscle thickness, masseter (cm)	M ± SD	1.02 ± 0.28	1.38 ± 0.15	*p* ≤ 0.01
	CA (MVC, electrical)	Muscle thickness, temporalis (cm)	M ± SD	0.83 ± 0.19	0.72 ± 0.17	*p* = 0.18
Ribeiro et al., 2017a	CA (transducer)	Bite force (N)	M ± SD	89.8 ± 25.50	157.9 ± 77.1 0	*p* ≤ 0.01
Rodrigues Ribeiro et al., 2019	CA (transducer)	Bite force (N)	M ± SD	13.4 ± 6.50	N/A	N/A
da Silva et al., 2019	CA (parafilm)	EMG, masseter	M ± SD	1.52 ± 0.22	1.03 ± 0.13	*p* = 0.08
	CA (parafilm)	EMG, temporalis	M ± SD	1.90 ± 0.44	0.98 ± 0.11	*p* ≤ 0.01
Chewing difficulties						
Adachi et al., 2012	CA (sweet potato)	MCD (s)	M	0.39^a^	0.31^a^	*p* ≤ 0.01
Anastassiadou et al., 2002	SR (Quest)	CD	Prevalence	39%	N/A	N/A
Bakke et al., 2011	SR (Quest)	CD	M ± SD	0.9 ± 1.0	0.0 ± 0.0	*p* ≤ 0.01
	CA (apple)	ME (s)	M ± SD	67.6 ± 57.8	34.4 ± 4.2	*p* = 0.10
	CA (gum)	MP (weight loss, %)	M ± SD	24.0 ± 11.5%	33.5 ± 3.8%	*p* ≤ 0.01
Baram et al., 2020	CA (apple)	ME (s)	M ± SD	28.4 ± 13.5	N/A	N/A
Baram et al., 2021	SR (Quest)	CD	M ± SD	0.7 ± 0.5	N/A	N/A
Karlsson et al., 1992	CA (peanut)	MCD (s)	M ± SD	0.50 ± 0.13	N/A	N/A
Massimo et al., 2020	CA (colour gum)	MP	M ± SD	3.2 ± 0.4	3.5 ± 0.8	NS
	SR (Quest)	CD	M ± SD	1.1 ± 1.0	0.8 ± 0.8	NS
Nakayama et al., 2004	SR (Quest)	CD	Prevalence	28%	6%	*p* ≤ 0.05
Persson et al., 1992	SR (Quest)	CD	Prevalence	33.3%	3.4%	NS
Ribeiro et al., 2017a	CA (Optocal cube)	MP (particle size, mm)	M ± SD	5.7 ± 0.9	4.2 ± 1.1	*p* ≤ 0.01
	CA (Optocal cube)	MCD (s)	M ± SD	0.77 ± 0.16	0.61 ± 0.10	*p* ≤ 0.05
Ribeiro et al., 2017b	CA (Optocal cube)	ME (weight loss, %)	M ± SD	7.0 ± 9.8%	13.0 ± 11.3%	*p* ≤ 0.05
Rodrigues Ribeiro et al., 2019	CA (Optocal cube)	MP (particle size, mm)	M ± SD	5.8 ± 1.1	N/A	N/A
	CA (Optocal cube)	MCD (s)	M ± SD	0.61 ± 0.14	N/A	N/A
van Stiphout et al., 2018	SR (Quest)	CD	Prevalence	29.7%	4.1%	*p* ≤ 0.01
Wooten Watts et al., 1999	SR (Quest)	CD	Prevalence	19%	6%	*p* ≤ 0.05
Unspecified TMD						
Bakke et al., 2011	SR + CA (NOT‐S)	U‐TMD	M ± SD	5.5 ± 2.9	0.7 ± 0.0	*p* ≤ 0.01
Baram et al., 2020	SR + CA (NOT‐S)	U‐TMD	M ± SD	3.1 ± 2.0	N/A	N/A
Baram et al., 2021	SR (NOT‐S)	U‐TMD	M ± SD	1.6 ± 1.2	N/A	N/A
Tavares et al., 2021	SR + CA (RDC/TMD)	U‐TMD	Prevalence	61.7%	N/A	N/A
Baumann et al., 2020	SR + CA (Helkimo)	U‐TMD	Prevalence	37.1%	2.4%	N/A
Choi et al., 2021	History (ICD‐10)	U‐TMD	Prevalence	1.0%	0.6%	*p* ≤ 0.05
Da Costa Silva et al., 2015	SR + CA (RDC/TMD)	U‐TMD	Prevalence	20.3%	N/A	N/A
Silva et al., 2016	SR + CA (RDC/TMD)	U‐TMD	Prevalence	23.8%	N/A	N/A
Massimo et al., 2020	SR + CA (NOT‐S)	U‐TMD	M ± SD	4.5 ± 2.3	1.1 ± 1.1	*p* ≤ 0.01
Non‐painful TMD						
Persson et al., 1992	SR (Quest)	NP‐TMD (Joint function)	Prevalence	40%	50%	NS
Verhoeff et al., 2018	SR (DC/TMD)	NP‐TMD (locks)	Prevalence	12.3%	18.3%	NS
Wooten Watts et al., 1999	SR (Quest)	NP‐TMD (sounds)	Prevalence	27%	17%	*p* ≤ 0.05
Sensory disturbances						
Bakke et al., 2011	CA	OS (response time, s)	M ± SD	8.6 ± 6.5	5.9 ± 3.1	*p* = 0.10
Bakke et al., 2011	CA	OS (identifications)	M ± SD	0.6 ± 0.6	0.8 ± 0.2	*p* = 0.20
Bruxism						
Abe et al., 2013	CA (PSG)	Bruxism (SB); RMMA	Episode index	0.52	0.00	*p* ≤ 0.01
	CA (PSG)	Bruxism (SB); RMMA	Burst index	1.94	0.00	*p* ≤ 0.01
Bonenfant et al., 2016	SR (Quest)	Bruxism (?); OHS	Prevalence	4.8%	N/A	N/A
Kwak et al., 2009	CA (Observe)	Bruxism (AB)	Prevalence	2.2%	N/A	N/A
Persson et al., 1992	SR (Quest)	Bruxism (?)	Prevalence	56.7%	2.1%	NS
Verhoeff et al., 2018	SR (DC/TMD)	Bruxism (AB)	Prevalence	46.0%	9.1%	*p* ≤ 0.01
	SR (DC/TMD)	Bruxism (SB)	Prevalence	24.3%	8.3%	*p* ≤ 0.01
Wooten Watts et al., 1999	SR (Quest)	Involuntary jaw movement	Prevalence	16%	0%	*p* ≤ 0.05
	SR (Quest)	Bruxism (?)	Prevalence	30%	21%	NS

Abbreviations: %, percentage; ?, unknown; AB, Awake Bruxism; CA, Clinical Assessment; CC sEMG, Craniovercial Overall surface electromyography; CD, Chewing Difficulties; cm, centimetre; DC/TMD, Diagnostic Criteria for Temporomandibular Disorders; EMG, Electromyography; exp, experimental; ICD‐10, 10th edition of the internation statistical classification of Diseases and Related Health Problems; M, Mean; MCD, masticatory cycle duration; ME, Masticatory Efficiency; MM, Marginal Mean; mm, millimetres; MMO, maximum mouth opening; MP, Masticatory Performance; MVC, Maximum Voluntary Contraction; N, Newton; N/A, Not Applicable; NOT‐S, Nordic Orofacial Test‐Screening; NS, Not significant; observe, observation by caregivers; OHS, Oral Habit Score; OS, Oral Stereognosis; *p*, *p*‐value; PSG, Polysomnography; Quest, Questionnaire; RDC/TMD, Research Diagnostic Criteria for Temporomandibular Disorders; RMMA, Rhytmic Masticatory Muscle Activity; s, seconds; SB, Sleep bruxism; SD, standard deviation; SR, Self‐report; U‐TMD, Unspecified Temporomandibular Disorder.

^a^ Estimation because of reading figure.

^b^ Within PD patients the ridigity group.

^c^ Within PD patients the tremor group.

^d^ Measured from lip to lip.

Nine studies analysed the amplitude of jaw movements (Albuquerque & da Silva, 2016; Bakke et al., 2011; Bandini et al., 2016; Baram et al., 2020; Katsikitis & Pilowsky, 1996; Robertson et al., 2001, 2011; Rodrigues Ribeiro et al., 2019). The within‐group results showed a range of 17.5 mm (Albuquerque & da Silva, 2016) to 49.2 (±6.9)mm (Baram et al., 2020) for the maximum mouth opening. The results of the between‐group comparisons, available for six studies, were largely similar (Adachi et al., 2012; Albuquerque & da Silva, 2016; Bakke et al., 2011; Bandini et al., 2016; Robertson & Hammerstad, 1996). Five studies found smaller amplitudes of jaw movements in patients with PD compared to controls (Adachi et al., 2012; Albuquerque & da Silva, 2016; Bakke et al., 2011; Robertson & Hammerstad, 1996). In contrast, Bandini et al. (2016) did not report significant differences (Bandini et al., 2016); however, they did report a smaller normalized range of opening in PD patients than in controls. Albuquerque and da Silva (2016) also included a control group, but they did not statistically analyse whether a difference was present (Albuquerque & da Silva, 2016). Nevertheless, they reported more deviation (i.e., unsymmetrical opening of the mouth) in patients with PD than in controls.

Only two studies analysed the self‐reported difficulties PD patients experience during jaw movements (Persson et al., 1992; Wooten Watts et al., 1999). Both studies included a control group. The between‐group results showed no significant group difference in the self‐reported difficulties patients experience with jaw movements compared to controls.

In total, seven studies investigated the velocity of jaw movements (Adachi et al., 2012; Adams et al., 2004; Albuquerque & da Silva, 2016; Bandini et al., 2016; Karlsson et al., 1992; Robertson et al., 2001; Robertson & Hammerstad, 1996), and all but one study included a control group (Karlsson et al., 1992). The within‐group results showed a range of 94.9 (±33.4)mm/s to 188 (±36)mm/s during the opening. Besides, the between‐group results showed significantly slower movements in PD patients than in controls (Adachi et al., 2012; Adams et al., 2004; Albuquerque & da Silva, 2016; Bandini et al., 2016; Robertson et al., 2001; Robertson & Hammerstad, 1996). Although Albuquerque and da Silva (2016) did include a control group and found slower movements in PD patients than in controls, no statistical analysis was performed (Albuquerque & da Silva, 2016). Only Robertson and Hammerstad (1996) found significantly faster jaw movements in PD patients than in the control group.

In conclusion, limited jaw movements and slower velocity of jaw movements are more prevalent in patients with PD than in controls.

#### 3.3.2 Maximum muscle output

Four studies analysed the maximum muscle output in patients with PD (Table 3; da Silva et al., 2019; Donizetti Verri et al., 2019; Rodrigues Ribeiro et al., 2019). The within‐group results showed varying results for bite force, ranging between 13.4 ± 6.5 N and 164 ± 96 N; and for muscle thickness, ranging between 0.7 ± 0.2 cm and 1.0 ± 0.3 cm. Between‐group results were reported in three studies that included a control group (da Silva et al., 2019; Donizetti Verri et al., 2019). Patients with PD had significantly lower maximum bite force than controls. Furthermore, the m. masseter is thinner in PD patients than in controls during rest and maximum voluntary contraction. However, the m. temporalis was thicker in PD patients than in controls during rest (Donizetti Verri et al., 2019). Only during maximum voluntary contraction, no statistically significant difference was found. Finally, the study of da Silva et al. (2019) showed significantly stronger electromyographic signals of the masticatory muscles during experimental chewing in PD patients than in controls.

In conclusion, the masticatory muscles of patients with PD tend to be thinner than those of controls. Although the bite force is lower in PD patients than in controls, the electromyographic signals tend to be higher during eating in PD patients than in controls.

#### 3.3.3 Chewing difficulties

In total, eight studies analysed self‐reported chewing difficulties in patients with PD (Table 3; Anastassiadou et al., 2002; Bakke et al., 2011; Baram et al., 2021; Massimo et al., 2020; Nakayama et al., 2004; Persson et al., 1992; van Stiphout et al., 2018; Wooten Watts et al., 1999). The within‐group results showed prevalences that varied between 19 and 39%. For the analysis of the between‐group results, six studies that included a control group were available (Bakke et al., 2011; Massimo et al., 2020; Nakayama et al., 2004; Persson et al., 1992; van Stiphout et al., 2018; Wooten Watts et al., 1999). Four of these studies reported significantly higher prevalences of chewing difficulties in PD patients than in controls (Bakke et al., 2011; Nakayama et al., 2004; van Stiphout et al., 2018; Wooten Watts et al., 1999). Although Persson et al. (1992) found no significant difference between the two groups, more chewing difficulties in PD patients were found in the ON‐phase (Margaretha Persson et al., 1992). Only Massimo et al. (2020) reported the same results for both groups (Massimo et al., 2020).

In addition to the prevalence of chewing difficulties, six studies reported difficulties in masticatory efficiency (i.e., the time or amount of chewing cycles needed to reduce the size of a specific food particle to be able to swallow it) and performance (i.e., an objective parameter which measured the size of food particles after a standard number of chewing cycles or the weight loss of gum [%]; Bakke et al., 2011; Baram et al., 2020; Massimo et al., 2020; Ribeiro et al., 2017a, 2017b; Rodrigues Ribeiro et al., 2019). The within‐group results demonstrated that patients with PD need 30 (±13.5) up to approximately 67 (±60) seconds to swallow a piece of apple (Bakke et al., 2011; Baram et al., 2020). Besides, masticatory performance analysed by means of weight loss ranged between 7.0 (±9.8) and 24.0 (±11.5)% weight loss of gum or optical cube (Bakke et al., 2011; Ribeiro et al., 2017a, 2017b). Finally, particle sizes were approximately 5.8 mm after chewing (Rodrigues Ribeiro et al., 2019). For the between‐group results, four studies that included a control group were available (Bakke et al., 2011; Massimo et al., 2020; Ribeiro et al., 2017a, 2017b). Bakke et al. (2011) found significantly worse masticatory performance in PD patients as compared to controls. Also, the efficiency was worse, albeit not significantly different (Bakke et al., 2011). Also, both studies of Ribeiro et al., (Ribeiro et al., 2017a, 2017b) found significantly worse masticatory performance in PD patients than in controls (Ribeiro et al., 2017a, 2017b). Only Massimo et al. (2020) found the same results for both groups.

Four studies investigated the masticatory cycle duration (Adachi et al., 2012; Karlsson et al., 1992; Rodrigues Ribeiro et al., 2019). The within‐group results ranged between 0.40 and 0.77 seconds per cycle. Only two studies included a between‐group analysis, which showed a significantly longer cycle duration during chewing in PD patients than in controls (Adachi et al., 2012).

In conclusion, patients with PD have more difficulties with chewing than controls.

#### 3.3.4 Unspecified TMD

In total, nine studies examined unspecified TMD (viz., no clear distinction was made between TMD pain or dysfunction) in patients with PD (Table 3; Bakke et al., 2011; Baram et al., 2020; Baram et al., 2021; Baumann et al., 2020; Choi et al., 2021; Da Costa Silva et al., 2015; Massimo et al., 2020; Silva et al., 2016; Tavares et al., 2021). The within‐group results showed prevalences that varied between 1.1 and 61.7%. The between‐group results revealed, in all three studies, significantly more unspecified TMD in PD patients than in controls (Bakke et al., 2011; Choi et al., 2021; Massimo et al., 2020).

In conclusion, in studies in which no distinction was made between pain or dysfunction, unspecified TMD is more prevalent in patients with PD than in controls.

#### 3.3.5 Non‐painful TMD

Only three studies examined non‐painful TMD (Persson et al., 1992; Verhoeff et al., 2018; Wooten Watts et al., 1999), of which all three included a control group (Table 3). Wooten Watts et al. found significantly more joint sounds in patients with PD than in controls. However, Verhoeff et al. (2018) and Persson et al. (1992) found no significant difference between both groups in the prevalence of having locks and impaired jaw function, respectively (Margaretha Persson et al., 1992; Merel C. Verhoeff et al., 2018).

All three studies examined different non‐painful TMD symptoms. Therefore, no conclusion can be drawn based on these studies.

#### 3.3.6 Sensory disturbances

Only one study investigated sensory disturbances in patients with PD (Table 3; Bakke et al., 2011). Bakke et al. (2011) reported that the time to recognize and discriminate shapes was slower in patients with PD compared to controls. However, this difference was not statistically significant. Besides, the number of positive identifications was almost the same in both groups (Bakke et al., 2011).

No strong conclusion can be drawn based on a single study which suggests that patients with PD may have orofacial sensory disturbances.

#### 3.3.7 Bruxism

Six articles investigated bruxism in patients with PD (Table 3; Abe et al., 2013; Bonenfant et al., 2016; Kwak et al., 2009; Persson et al., 1992; Verhoeff et al., 2018; Wooten Watts et al., 1999). The within‐group results showed prevalences that varied between 2 and 57%. Besides, four articles included a control group and were thus suitable to analyse between‐group results (Abe et al., 2013; Persson et al., 1992; Verhoeff et al., 2018; Wooten Watts et al., 1999). Only one of these studies differentiated between the two circadian forms of bruxism, i.e., awake‐related and sleep‐related, and found a significantly higher prevalence for both awake bruxism and sleep bruxism in patients with PD compared to controls (Verhoeff et al., 2018). Furthermore, Wooten Watts et al. (1999) found a higher prevalence of bruxism in patients with PD than in controls; however, this difference was not statistically significant. The same study did find a higher prevalence of involuntary movements of the jaw and/or mouth in patients with PD than in controls (Wooten Watts et al., 1999). Persson et al. (1992) did not find a significant difference between PD patients and controls; however, the prevalence of bruxism in PD patients was approximately 50% higher than in the control group. Moreover, Abe et al. (2013) found higher Rythmic Masticatory Muscle Activity (RMMA) indices for sleep bruxism episodes and bursts in patients with PD compared to controls. Finally, a case study described a case of a woman with PD who developed bruxism and tooth wear after using a (nowadays unusually high) dosage of 4.5grams of levodopa. Because the beneficial effect on her motor symptoms was so strong, no other medical treatment was used. Her teeth were protected by means of splint therapy, which stopped progressing the wear (Table 4; Magee, 1970).

**TABLE 4:** Results of case studies and case series, in PD patients

Article	Number of cases	Age (in years)	Gender (%female)	Variable	Outcome
Coon & Laughlin, 2012	1	65	100%	BMD	6 Weeks after starting 25/100 mg carbidopa/levodopa BMD started; after discontinuation carbidopa/levodopa the symptoms disappeared in 2 weeks' time. Pramipexol in higher dosages (1.5 mg) was prescribed with the release of PD symptoms, but without BMD
Ford et al., 1996	5	66 (M)	60%	BMD	All cases experienced burning sensation and oral discomfort of the oral cavity, gums and/or face
Magee, 1970	1	53	100%	Bruxism	After using levodopa for several months, where the dosage was slowly built up to 4.5gram, PD symptoms were acceptable. However, after five months of therapy, bruxism occurred with severe tooth wear as consequence. A cast was made to protect her teeth for further development of tooth wear, but did not adjust or stopped the levodopa therapy
Minagi et al., 1998	1	71	100%	TMD pain	Experienced frequent and excessive involuntary movement to the right side of the TMJ, with her mandible caused pain at the location of her TMJ. After making an appliance that restricted her movement, the pain diminished after one week

Abbreviations: %, Percentage; BMD, Burning Mouth Disorder; M, Mean; mg, milligram; PD, Parkinson'’s Disease; TMD, Temporomandibular Disorder; TMJ, Temporomandibular Joint.

In conclusion, the prevalence of both circadian forms of bruxism in PD patients appears to be higher than in controls.

### 3.4 Associated factors with orofacial pain and/or dysfunction complaints in PD patients

#### 3.4.1 Gender

Four studies analysed whether gender is associated with orofacial pain and/or dysfunction complaints in patients with PD (Table 5 Bonenfant et al., 2016; Clifford et al., 1998; Da Costa Silva et al., 2015; O'Neill et al., 2021). Da Costa Silva et al. (2015) showed a higher prevalence of unspecified TMD in females compared to males. However, this finding was not statistically analysed. Furthermore, O'Neill et al. (2021) found a higher prevalence of orofacial pain in females compared to males (O'Neill et al., 2021). In addition, Bonenfant et al. found a higher prevalence of BMD in males than in females. In contrast, Clifford et al. (1998) found a significantly higher prevalence of BMD in females than in males.

**TABLE 5:** Results for the possible associated PD‐related variables (viz., gender, disease duration, disease severity, and medication usage) with orofacial pain and dysfunction complaints in patients with PD, compared with healthy controls

Associated factors			
	Gender		
Article	Males	Females	*p*‐value
Bonenfant et al., 2016	10.3% BMD	4.3% BMD	N/A
Clifford et al., 1998	10 BMD	17 BMD	*p* ≤ 0.01
Da Costa Silva et al., 2015	41.7% prevalence of unspecified TMD	58.3% prevalence of unspecified TMD	N/A
O'Neill et al., 2021	5.9% prevalence orofacial pain	10.4% orofacial pain prevalence	N/A

Article	Variable	PD
Disease duration		
Bakke et al., 2011	↓ Unspecified TMD when the duration of the disease is longer	*p* ≤ 0.05
	↑ Self‐reported chewing difficulties when the duration of the disease is longer	*p* ≤ 0.05
Baram et al., 2021	↓ Unspecified TMD when the duration of the disease is longer	*p* = 0.5
	Masticatory ability is not associated with the duration of the disease	NS
Bonenfant et al., 2016	Median disease duration is equal for BMD and non‐BMD group	NS
O'Neill et al., 2021	↑ Disease duration when BMD is present (5.8 ± 7.3) than without BMD (2.9 ± 1.9)	*p* ≤ 0.05
Disease severity		
Bakke et al., 2011	↓ Orofacial function when disease severity is worse	*p* ≤ 0.01
	↑ Self‐reported chewing difficulties when disease severity is worse	*p* ≤ 0.01
	↑ Sensory disturbances when disease severity is worse	*p* ≤ 0.01
	No association between the tenderness of jaw elevator muscles and disease severity	*p* = 0.7
	↑ Sensory disturbances (recognition) when disease severity is worse	*p* ≤ 0.05
	No association between mouth opening and severity of the disease	NS
Baram et al., 2020	↑ Orofacial dysfunction when disease severity is worse	*p* ≤ 0.01
Baram et al., 2021	↓ Orofacial function (NOT‐S) when disease severity is worse	*p* ≤ 0.01
	↓ Masticatory ability when disease severity is worse	*p* ≤ 0.05
Bonenfant et al., 2016	↑ Median H&Y scale when BMD is present (3) when compared to no BMD (2.5)	NS
Chen et al., 2019	↑ Incidence of TMD during the first and second year of the disease	*p* ≤ 0.05
Da Costa Silva et al., 2015	No association between TMD and disease severity	NS
Massimo et al., 2020	No association between masticatory efficiency (gum chewing) and disease severity	NS
O'Neill et al., 2021	↑ Prevalence of orofacial pain when disease severity (subdomains MDS‐UPDRS) is worse	?
Rodríguez‐Violante et al., 2017	↑ Prevalence of orofacial pain scores when disease severity is worse	*p* ≤ 0.01
van Stiphout et al., 2018	↑ Chewing and biting difficulties when disease severity is worse	*p* ≤ 0.05
Medication usage		
Bonenfant et al., 2016	↓ Mean LEDD score when BMD is present (630.1 mg/day) when compared to no BMD (653.9 mg/day)	NS
Karlsson et al., 1992	↓ Slower movements and mandibular displacements after ON‐phase compared to OFF‐period	*p* ≤ 0.05
O'Neill et al., 2021	↑ Median LEDD score when BMD is present (465 mg/day) when compared to no BMD (400 mg/day)	*p* ≤ 0.01*
	↑ Median LEDD score when grinding pain is present (462.5 mg/day) when compared to no pain (400 mg/day)	*p* ≤ 0.01*
Rodriguez Ribeiro et al., 2019 [MF]	↑ Bigger range of jaw motion (lateral deviation, protrusion, laterotrusion) during ON‐period compared to OFF‐period	*p* ≤ 0.01
	MMO during ON‐period (31.0 ± 5.6 mm) compared to MMO during OFF‐period (31.3 ± 3.1 mm)	NS
	↑ Higher maximum bite force during ON‐period compared to OFF‐period	*p* ≤ 0.01
	↑ Better masticatory performance ON‐period compared to OFF‐period	*p* ≤ 0.01
Robertson & Hammerstad, 1996	↑ MMO during the ON‐period compared to the OFF‐period	*p* ≤ 0.01
	↓ EMG pattern during clenching in the OFF‐period compared to ON‐period	N/A
Robertson et al., 2001	↑ Jaw opening velocity and MMO during ON‐period compared to OFF‐period	N/A
Robertson et al., 2011	↑ Jaw opening and closing velocity during ON‐period compared to OFF‐period	N/A

Abbreviations: %, percentage; ??, unknown; ↑, higher; ↓, lower; BMD, Burning Mouth Disorder; EMG, Electromyography; H&Y, Hoehn & Yahr Scale; LEDD, Levodopa Equivalent Daily Dosages; MDS‐UPDRS, Movement Disorders Society Unified Parkinson Disease Rating Scale; mg/day, milligrams per day; MMO, Maximum Mouth Opening; N/A, Not Applicable; NOT‐S, Nordic Orofacial Test Screening; NS, Not Statistically Different; OFF, Dopaminergic therapy is working suboptimal; ON, dopaminergic therapy works optimal; *p*, *p*‐value; TMD, Temporomandibular Disorder.

* After Bonferroni correction statistical significance dissapeared.

In conclusion, the female gender appears to be associated with orofacial pain and/or dysfunction in PD patients, although the available evidence is not fully aligned.

#### 3.4.2 Disease duration

Four studies analysed whether disease duration is associated with orofacial pain and/or dysfunction in PD patients (Table 5; Bakke et al., 2011; Baram et al., 2021; Bonenfant et al., 2016; O'Neill et al., 2021). O'Neill et al. (2021) found significantly more BMD with longer disease duration. However, Bonenfant et al. (2016) found the same median disease duration for PD patients with and without BMD (Bonenfant et al., 2016). Moreover, Baram et al. (2021) found no correlation between masticatory efficiency and unspecified TMD on the one hand, and disease duration on the other (S Baram et al., 2021). Finally, lower unspecified TMD prevalences and higher self‐reported chewing difficulties were found in the study by Bakke et al. (2011), when the disease duration was longer (Bakke et al., 2011).

In conclusion, disease duration seems to be associated with self‐reported chewing difficulties and the presence of BMD complaints.

#### 3.4.3 Disease severity

Nine studies analysed whether disease severity is associated with orofacial pain and/or dysfunction (Table 5; Bakke et al., 2011; Baram et al., 2020; Baram et al., 2021; Bonenfant et al., 2016; Chen et al., 2019; Da Costa Silva et al., 2015; Massimo et al., 2020; O'Neill et al., 2021; Rodríguez‐Violante et al., 2017; van Stiphout et al., 2018). Of these, six studies found significantly more orofacial pain and/or dysfunction, namely, orofacial pain, unspecified TMD, non‐painful TMD, self‐reported chewing difficulties, masticatory efficiency, and sensory disturbances, when disease severity was worse (Bakke et al., 2011; Baram et al., 2020; Baram et al., 2021; O'Neill et al., 2021; Rodríguez‐Violante et al., 2017; van Stiphout et al., 2018). On the contrary, three studies found no correlation between disease severity and orofacial pain and/or dysfunction, namely the maximum mouth opening, myalgia, unspecified TMD, and masticatory efficiency on the one hand and disease severity on the other (Bakke et al., 2011; Da Costa Silva et al., 2015; Massimo et al., 2020).

In conclusion, prevalences of orofacial pain, unspecified TMD, non‐painful TMD, self‐reported chewing difficulties, masticatory efficiency, and sensory disturbances are suggested to be higher when the severity of PD is worse.

#### 3.4.4 Medication usage

Seven articles analysed whether medication usage is associated with orofacial pain and/or dysfunction (Table 5; Bonenfant et al., 2016; Karlsson et al., 1992; O'Neill et al., 2021; Robertson et al., 2001, 2011; Robertson & Hammerstad, 1996; Rodrigues Ribeiro et al., 2019). Bonenfant et al. (2016) did not find an association between the LEDD (i.e., Levodopa Equivalent Daily Dosage) and the prevalence of BMD (Bonenfant et al., 2016). However, O'Neill et al. (2021) found higher median LEDD scores when BMD was present compared to no BMD in PD patients (O'Neill et al., 2021). Moreover, O'Neill et al. (2021) found a higher median LEDD score when orofacial pain was present during grinding, compared to no pain.

Rodriguez Ribeiro et al. (2019) did not find significant associations between the maximum mouth opening during the ON‐period and the OFF‐period (Rodrigues Ribeiro et al., 2019). However, they found a larger range of jaw movements, higher maximum bite force, and better masticatory performance during the ON‐period than the OFF‐period. In contrast, Robertson & Hammerstad (1996) (Robertson & Hammerstad, 1996), Robertson et al. (2001), and Robertson et al. (2011)(Robertson et al., 2011) found a larger maximum mouth opening during the ON‐period compared to the OFF‐period; however, only Robertson and Hammerstad (1996) found significant differences between both phases (Robertson & Hammerstad, 1996). Finally, when analysing the velocity of the jaw movements, slower movements were found during the OFF‐period compared to the ON‐period in all three studies.

In conclusion, when dopaminergic therapy is working optimally (i.e. in the ON state), fewer orofacial pain and/or dysfunction complaints, such as the presence of BMD, limited jaw movements, slower jaw movements and masticatory performance, seem to be present (Table 5).

## 4 DISCUSSION

This scoping review aimed to give a broad overview of the relevant literature on the prevalence of orofacial pain and/or dysfunction in patients with PD and, when available, the comparison with controls. Furthermore, we aimed to see which patient‐related characteristics are associated with orofacial pain and/or dysfunction in PD patients and to generate hypotheses for future research on this topic. The majority of the studies showed that orofacial pain and/or dysfunction in the orofacial area are more common in PD patients than in healthy persons. Moreover, some studies found a correlation between, on the one hand, disease severity and other disease‐related factors (e.g. medication usage) and, in contrast, orofacial pain and/or dysfunction.

In this scoping review, orofacial pain and TMD pain were more common in PD patients than in healthy controls. Pain, in general, is a common problem in patients with PD. Various types of pain have been described in PD patients (e.g. musculoskeletal pain, neuropathic pain, central pain), and several classifications and diagnostic tools have been proposed (Chaudhuri et al., 2011; Cury et al., 2016). The exact mechanisms that are responsible for a higher prevalence of pain in PD are largely unknown. However, it has been suggested that pain thresholds are lower in patients with PD during the OFF‐phase and that PD patients, therefore, experience more pain (Tai & Lin, 2020). Recently, a validated classification system was published to analyse whether pain in PD patients is related to the disease itself, or whether it could be non‐PD related pain (Mylius et al., 2021). Besides, in this classification system, a distinction was made between the three mechanisms causing pain: nociceptive, neuropathic and neuroplastic pain. This is an important step towards understanding complicated pain mechanisms in PD patients related or unrelated to the disease itself. Besides, motor symptom fluctuations and dopaminergic medication can influence pain intensity. Whether dopaminergic medication has an antinociceptive effect or a modulatory effect on pain perception is still unclear (Tai & Lin, 2020). In this scoping review, results concerning dopaminergic medication and pain are ambiguous: on the contrary, a positive association was found between the use of dopaminergic medication and pain, and on the other hand, some patients reported that the pain started after starting levodopa treatment. Moreover, in the case series more pain was experienced in the OFF state. Although the evidence level of the case series is low, this confirms the hypothesis that dopamine may positively affect pain mechanisms. Furthermore, after initiation of levodopa therapy, the pain thresholds of PD patients are found to be significantly, albeit temporarily, raised in comparison to controls. In contrast to the suggestion that dopamine could alleviate PD symptoms, such as pain, it is also possible that the progression of motoric PD symptoms is worse and thus accountable for more pain, despite medication usage. Unfortunately, only limited high‐quality data is available on this topic. Hence, the results suggesting that dopaminergic therapy may reduce orofacial pain should be interpreted with caution. Besides, it is necessary to allow for the possibility that in PD patients also other motor symptoms could occur in the orofacial region. For example, bruxism after using levodopa could be interpreted as dyskinesia or vice versa. Therefore, it is recommended for future research, to critically investigate the possibility of shared characteristics of these orofacial motor symptoms. Future research should include medication dose as a parameter, to analyse the association between dopaminergic therapy and orofacial motor symptoms in more detail as well as to assess the effects of medication usage on the possible fluctuating character of pain in the orofacial region.

The majority of the studies included in this scoping review showed that PD patients have limited jaw movements in terms of maximum mouth opening and velocity of movements, and that chewing difficulties and unspecified TMD were also more common in PD patients than in controls. It is known that this patient group has reduced oral health (van Stiphout et al., 2018), which could yield impaired chewing function due to loss of teeth or dental pain. It is important to mention that the consequences of impaired chewing ability are suggested to be farther reaching than difficulties with eating only. For example, impaired chewing abilities may be associated with cognitive dysfunctionc (Weijenberg et al., 2019). Besides, muscle force is lower in PD patients than in healthy controls, which was also found in the orofacial area (Donizetti Verri et al., 2019). An impairment of oral function could limit, for example, social activities such as having dinner with friends or conversations with people (Verhoeff et al., 2022). Therefore, it is important to develop strategies to improve these limitations. Because of the clinical heterogeneity of PD and its progressive nature, this issue is complex to study. Nevertheless, some studies have assessed therapeutic strategies to reduce the limitations in jaw movements (e.g. physiotherapy, Deep Brain Stimulation [DBS] or insertion of a well‐fitting removable prosthesis) and showed promising results (Baram et al., 2020; Katsikitis & Pilowsky, 1996; Ribeiro et al., 2017a, 2017b; Robertson et al., 2001). For example, two studies found a significant improvement in masticatory efficiency and performance when instruction was given, or when a new well‐fitting removable prosthesis was made (Baram et al., 2020; Ribeiro et al., 2017a, 2017b). In addition, the studies of Baram et al. (2020), Katsikitis and Pilowsky (1996) and Robertson et al. (2001) have shown improvement in opening and closing velocity through various therapies (e.g. physiotherapy and DBS). Therefore, using such treatments, PD patients' oral function can be ameliorated, and hence their oral health‐related quality of life can be improved (Verhoeff et al., 2022). (Oral) health practitioners need to acknowledge this worrisome issue that exceeds beyond the oral cavity. Therefore, inter‐multidisciplinary approaches should be considered when treating patients with PD (Lobbezoo & Aarab, 2021).

Bruxism was only described in six articles. In the majority of these articles, the probability level that bruxism is actually present (viz., according to the international bruxism consensus report; Lobbezoo et al., 2018) did not exceed a ‘possible’ bruxism diagnosis, i.e., based on self‐report. However, assumptions were made that bruxism is more prevalent in PD patients than in healthy controls because of the hypothesis that, amongst others: (i) the dopaminergic system plays a role in both PD and bruxism; (ii) the prodromal phase of PD shows comparable characteristics during sleep as bruxism; finally (iii) depression is more prevalent in PD patients and is also a risk factor for the presence of bruxism. More research with higher probability levels is needed to determine whether this hypothesis can be accepted and whether other factors are involved, such as disease‐related variables (viz., disease duration, medication usage).

### 4.1 Limitations and strengths

This scoping review has several limitations. First, patients in all included studies are between 60 and 70 years of age, the average duration of the disease did not exceed 15 years, and the severity of the disease was in the majority of the included articles not higher than Hoehn & Yahr scale three (i.e. on a 5‐point ordinal scale). Hence, because PD is a life‐long condition and because of the progressive nature of PD, the severity of the disease was probably relatively mild. Because suggestions are made that the severity of PD could be associated with pain and dysfunction in the orofacial area, one could reason that the reported findings represent an underestimation. Second, although this scoping review did not include a quality assessment, the majority of the included studies were of low to mid quality and were hampered by various sorts of bias (e.g. lack of transparency regarding the inclusion of participants or missing values; lacking characteristics of the study participants, such as dose of dopaminergic medication). Third, the methodology of the papers, their outcome variables and the amount of included articles per subject prevented us from performing a meta‐analysis. Therefore, this scoping review presented the data descriptively. Fourth, not every article described how PD patients received a PD diagnosis (i.e. which criteria were taken into consideration to set the PD diagnosis). To be able to compare research on PD patients, uniformity is recommended. Fifth, when PD was present unilaterally, none of the articles mentioned which side was affected. Because the affected side reflects the pathophysiology of the degeneration in the involved hemisphere, it is possible that orofacial pain and/or dysfunction are present on that affected side as well. The prime strength of this scoping review is that it was performed in collaboration with a medical librarian who performed a systematic and extensive search in multiple databases. Therefore, this review provides a comprehensive overview of the relevant literature on this topic and thus on the gaps in the literature to be filled by future research. Sixth, limited data are available on the influence of therapeutic options for PD patients on orofacial pain and dysfunction complaints. Therefore, in the current article, we chose to only focus on the presence of orofacial pain and dysfunction, and the possible influence of disease‐related factors.

### 4.2 Implications

Limited high‐quality studies are available on orofacial pain and dysfunction in PD patients. Notwithstanding, this literature review can serve to increase the awareness of health care providers of the problems that can be encountered in the orofacial area of PD patients. Further, it can assist to encourage collaboration between medicine and dentistry. Finally, based on the outcomes of this scoping review, new research can be designed, based on the gaps identified in the current literature on this topic.

## 5 CONCLUSIONS

In conclusion, orofacial pain and/or dysfunction is more prevalent in PD patients than in controls. Furthermore, in some studies, a correlation was found between, on the one hand, disease severity and other disease‐related factors (e.g. medication use) and, on the other hand, orofacial pain and/or dysfunction. Based on our findings, a number of hypotheses could be formulated: (i) orofacial pain and/or dysfunction is more prevalent in PD patients than in controls; (ii) disease duration and severity are associated with a higher prevalence of orofacial pain and worse orofacial function in patients with PD as compared to controls; (iii) medication, for example, dopaminergic therapy, reduces the prevalence of pain, raises pain thresholds (temporarily) and improves orofacial dysfunction in patients with PD. To test these hypotheses, we recommend designing a study that includes PD patients with a wide range of disease stages, from disease onset to advanced stages of the disease, to study whether disease duration and severity are associated with more orofacial pain and worse orofacial function. In addition, disease‐related factors (e.g. dose of dopaminergic medication) should be included in future studies to establish whether or not these factors can influence orofacial pain and/or dysfunction in PD patients. Furthermore, using validated and internationally approved diagnostic criteria (e.g. for TMD diagnosis the Diagnostic Criteria for Temporomandibular Disorders [DC/TMD]) is highly recommended to be able to, for example, compare the results of different studies. Finally, an interdisciplinary approach is recommended to overcome bias related to the field of interest (Lobbezoo & Aarab, 2021). Ultimately, this could contribute to improved, individualized and preferably preventive strategies aimed at reducing orofacial pain, dysfunction and its consequences in PD patients.

## DISCLOSURE

The researchers did not receive any grant from a commercial, public or not‐for‐profit funding agency to perform this study.

## AUTHOR CONTRIBUTIONS

MV, MK and FL conceived of the present idea. RdV performed the search. MV, ST and DE screened the results and extracted the data from the papers. All authors contributed to the clinical perspective and final review of this manuscript.

## CONFLICT OF INTEREST

The authors declare no conflicts of interest.

## Supporting information


Table S1
