# Supplementary material for: Orofacial pain and dysfunction in patients with Parkinson's disease: A scoping review
Source: Eur J Pain. 2022 Sep 16;26(10):2036–59. doi: 10.1002/ejp.2031 (PMC9826258; doi:10.1002/ejp.2031)
Supplement: Supplementary file 1 — Table S1 [file EJP-26-2036-s001.docx]

**TableS1**: Search strategy

**PubMed Session Results (20 Jan 2022)**

| Search | Query | Items found |
| --- | --- | --- |
| #9 | **#7 OR #8** | 2,860 |
| #8 | **#1 AND #4** | 256 |
| #7 | **#5 OR #6** | 2,661 |
| #6 | **#1 AND #3** | 1,617 |
| #5 | **#1 AND #2** | 1,270 |
| #4 | **(("Quality of Life"[Mesh] OR "quality of life"[tiab] OR "life qualit*"[tiab] OR "living qualit*"[tiab] OR "quality of living"[tiab] OR "Activities of Daily Living"[Mesh] OR "activities of daily living"[tiab] OR "activity of daily living"[tiab] OR "activities of daily life"[tiab] OR "activity of daily life"[tiab] OR "daily living activit*"[tiab] OR "daily life activit*"[tiab] OR "adl"[tiab] OR "chronic limitation of activity"[tiab] OR "self care*"[tiab] OR "Health Status"[Mesh] OR "health status"[tiab] OR "level of health"[tiab] OR "health level*"[tiab] OR "qol"[tiab] OR "hrql"[tiab] OR "hrqol"[tiab]) AND (oral[tiab])) OR "OHRQoL"[tiab]** | 29,820 |
| #3 | **"Dyskinesias"[Mesh:NoExp] OR "Mastication"[Mesh] OR "Facial Pain"[Mesh] OR "Facial Neuralgia"[Mesh] OR "Musculoskeletal Pain"[Mesh:NoExp] OR "Myalgia"[Mesh] OR "Arthralgia"[Mesh:NoExp] OR "Neuralgia"[Mesh:NoExp] OR "Burning Mouth Syndrome"[Mesh] OR "Craniomandibular Disorders"[Mesh] OR "Bruxism"[Mesh] OR "Dental Occlusion"[Mesh] OR "Malocclusion"[Mesh] OR "Tooth Wear"[Mesh] OR "oral function*"[tiab] OR "oral dyskinesia*"[tiab] OR "orofacial function*"[tiab] OR "orofacial dyskinesia*"[tiab] OR "mastication"[tiab] OR "chewing"[tiab] OR "tooth mobilit*"[tiab] OR "jaw mobilit*"[tiab] OR "mandibular mobilit*"[tiab] OR "tooth movement*"[tiab] OR "jaw movement*"[tiab] OR "mandibular movement*"[tiab] OR "orofacial pain"[tiab] OR "craniofacial pain"[tiab] OR "myofacial pain"[tiab] OR "facial pain"[tiab] OR "neuropathic pain"[tiab] OR "burning mouth"[tiab] OR "craniomandibular disorder*"[tiab] OR "cranio-mandibular disorder*"[tiab] OR "neuralgia"[tiab] OR "trigeminal"[tiab] OR "Tic Douloureux"[tiab] OR "temporomandibular joint dis*"[tiab] OR "temporo-mandibular joint dis*"[tiab] OR "temporomandibular dysfunction*"[tiab] OR "temporo-mandibular dysfunction*"[tiab] OR "temporomandibular disorder*"[tiab] OR "temporo-mandibular disorder*"[tiab] OR "TMJ dis*"[tiab] OR "TMD"[tiab]** | 192,191 |
| #2 | **"Oral Health"[Mesh] OR "Mouth Diseases"[Mesh] OR "Tooth Diseases"[Mesh] OR "Periodontal Prosthesis"[Mesh] OR "Periodontal Index"[Mesh] OR "Prosthodontics"[Mesh] OR "oral health"[tiab] OR "oral hygiene"[tiab] OR "dental"[tiab] OR "dentistry"[tiab] OR "mouth"[tiab] OR "tooth"[tiab] OR "teeth"[tiab] OR "jaw"[tiab] OR "jaws"[tiab] OR "periodont*"[tiab] OR "parodont*"[tiab] OR "Pyorrhea Alveolaris"[tiab] OR "periapical"[tiab] OR "gingiva*"[tiab] OR "gingivi*"[tiab] OR (("gum"[tiab] OR "gums"[tiab]) AND ("inflammat*"[tiab] OR "disease*"[tiab])) OR "caries"[tiab] OR "carious"[tiab] OR "edentulous"[tiab] OR "prosthes*"[tiab] OR "prosthetic*"[tiab] OR "prosthodont*"[tiab]** | 982,386 |
| #1 | **"Parkinsonian Disorders"[Mesh] OR "parkinson*"[tiab]** | 143,272 |

**Embase.com Session Results (20 Jan 2022)**

| Search | Query | Items found |
| --- | --- | --- |
| #10 | **#9 NOT ('conference abstract'/it OR 'conference review'/it)** | 4,695 |
| #9 | **#7 OR #8** | 6,093 |
| #8 | **#1 AND #4** | 692 |
| #7 | **#5 OR #6** | 5,547 |
| #6 | **#1 AND #3** | 2,045 |
| #5 | **#1 AND #2** | 4,009 |
| #4 | **(('quality of life'/exp OR 'quality of life':ab,ti,kw OR 'life qualit*':ab,ti,kw OR 'living qualit*':ab,ti,kw OR 'quality of living':ab,ti,kw OR 'daily life activity'/exp OR 'activities of daily living':ab,ti,kw OR 'activity of daily living':ab,ti,kw OR 'activities of daily life':ab,ti,kw OR 'activity of daily life':ab,ti,kw OR 'daily living activit*':ab,ti,kw OR 'daily life activit*':ab,ti,kw OR 'adl':ab,ti,kw OR 'chronic limitation of activity':ab,ti,kw OR 'self care*':ab,ti,kw OR 'health status'/exp OR 'health status':ab,ti,kw OR 'level of health':ab,ti,kw OR 'health level*':ab,ti,kw OR 'qol':ab,ti,kw OR 'hrql':ab,ti,kw OR 'hrqol':ab,ti,kw) AND (oral:ab,ti,kw)) OR 'oral health related quality of life'/exp OR 'OHRQoL':ab,ti,kw** | 45,365 |
| #3 | **'mastication'/exp OR 'face pain'/exp OR 'musculoskeletal pain'/de OR 'myofascial pain'/exp OR 'arthralgia'/exp OR 'neuralgia'/de OR 'burning mouth syndrome'/exp OR 'temporomandibular joint disorder'/exp OR 'bruxism'/exp OR 'tooth occlusion'/exp OR 'malocclusion'/exp OR 'jaw movement'/exp OR 'oral function*':ab,ti,kw OR 'oral dyskinesia*':ab,ti,kw OR 'orofacial function*':ab,ti,kw OR 'orofacial dyskinesia*':ab,ti,kw OR 'mastication':ab,ti,kw OR 'chewing':ab,ti,kw OR 'tooth mobilit*':ab,ti,kw OR 'jaw mobilit*':ab,ti,kw OR 'mandibular mobilit*':ab,ti,kw OR 'tooth movement*':ab,ti,kw OR 'jaw movement*':ab,ti,kw OR 'mandibular movement*':ab,ti,kw OR 'orofacial pain':ab,ti,kw OR 'craniofacial pain':ab,ti,kw OR 'myofacial pain':ab,ti,kw OR 'facial pain':ab,ti,kw OR 'neuropathic pain':ab,ti,kw OR 'burning mouth':ab,ti,kw OR 'craniomandibular disorder*':ab,ti,kw OR 'cranio-mandibular disorder*':ab,ti,kw OR neuralgia:ab,ti,kw OR trigeminal:ab,ti,kw OR 'Tic Douloureux':ab,ti,kw OR 'temporomandibular joint dis*':ab,ti,kw OR 'temporo-mandibular joint dis*':ab,ti,kw OR 'temporomandibular dysfunction*':ab,ti,kw OR 'temporo-mandibular dysfunction*':ab,ti,kw OR 'temporomandibular disorder*':ab,ti,kw OR 'temporo-mandibular disorder*':ab,ti,kw OR 'TMJ dis*':ab,ti,kw OR TMD:ab,ti,kw** | 282,483 |
| #2 | **'oral health related quality of life'/exp OR 'oral health status'/exp OR 'mouth disease'/exp OR 'periodontic device'/exp OR 'dental disease assessment'/exp OR 'prosthodontics'/exp OR 'oral health':ab,ti,kw OR 'oral hygiene':ab,ti,kw OR dental:ab,ti,kw OR dentistry:ab,ti,kw OR mouth:ab,ti,kw OR tooth:ab,ti,kw OR teeth:ab,ti,kw OR jaw:ab,ti,kw OR jaws:ab,ti,kw OR periodont*:ab,ti,kw OR parodont*:ab,ti,kw OR 'Pyorrhea Alveolaris':ab,ti,kw OR periapical:ab,ti,kw OR gingiva*:ab,ti,kw OR gingivi*:ab,ti,kw OR ((gum OR gums) NEAR/3 (inflammat* OR disease*)):ab,ti,kw OR caries:ab,ti,kw OR carious:ab,ti,kw OR edentulous:ab,ti,kw OR prosthes*:ab,ti,kw OR prosthetic*:ab,ti,kw OR prosthodont*:ab,ti,kw** | 1,181,125 |
| #1 | **'Parkinson disease'/exp OR 'parkinsonism'/exp OR parkinson*:ab,ti,kw** | 232,537 |

**Web of Science (Core Collection) Session Results (20 Jan 2022)**

| Search | Query | Items found |
| --- | --- | --- |
| #9 | **#7 OR #8** | 2,263 |
| #8 | **#1 AND #4** | 345 |
| #7 | **#5 OR #6** | 1,982 |
| #6 | **#1 AND #3** | 1,083 |
| #5 | **#1 AND #2** | 1,148 |
| #4 | **TS=((("quality of life" OR "life qualit*" OR "living qualit*" OR "quality of living" OR "activities of daily living" OR "activity of daily living" OR "activities of daily life" OR "activity of daily life" OR "daily living activit*" OR "daily life activit*" OR "adl" OR "chronic limitation of activity" OR "self care*" OR "health status" OR "level of health" OR "health level*" OR "qol" OR "hrql" OR "hrqol") AND (oral)) OR "OHRQoL")** | 29,779 |
| #3 | **TS=("oral function*" OR "oral dyskinesia*" OR "orofacial function*" OR "orofacial dyskinesia*" OR "mastication" OR "chewing" OR "tooth mobilit*" OR "jaw mobilit*" OR "mandibular mobilit*" OR "tooth movement*" OR "jaw movement*" OR "mandibular movement*" OR "orofacial pain" OR "craniofacial pain" OR "myofacial pain" OR "facial pain" OR "neuropathic pain" OR "burning mouth" OR "craniomandibular disorder*" OR "cranio-mandibular disorder*" OR "neuralgia" OR "trigeminal" OR "Tic Douloureux" OR "temporomandibular joint dis*" OR "temporo-mandibular joint dis*" OR "temporomandibular dysfunction*" OR "temporo-mandibular dysfunction*" OR "temporomandibular disorder*" OR "temporo-mandibular disorder*" OR "TMJ dis*" OR "TMD")** | 121,778 |
| #2 | **TS=("oral health" OR "oral hygiene" OR "dental" OR "dentistry" OR "mouth" OR "tooth" OR "teeth" OR "jaw" OR "jaws" OR "periodont*" OR "parodont*" OR "Pyorrhea Alveolaris" OR "periapical" OR "gingiva*" OR "gingivi*" OR (("gum" OR "gums") NEAR/3 ("inflammat*" OR "disease*")) OR "caries" OR "carious" OR "edentulous" OR "prosthes*" OR "prosthetic*" OR "prosthodont*")** | 653,348 |
| #1 | **TS=("parkinson*")** | 195,491 |

**Wiley / Cochrane Library Session Results (20 Jan 2022)**

| Search | Query | Items found |
| --- | --- | --- |
| #9 | **#7 OR #8** | 497 |
| #8 | **#1 AND #4** | 260 |
| #7 | **#5 OR #6** | 256 |
| #6 | **#1 AND #3** | 59 |
| #5 | **#1 AND #2** | 208 |
| #4 | **((((quality NEXT of NEXT life) OR (life NEXT qualit*) OR (living NEXT qualit*) OR (quality NEXT of NEXT living) OR (activities NEXT of NEXT daily NEXT living) OR (activity NEXT of NEXT daily NEXT living) OR (activities NEXT of NEXT daily NEXT life) OR (activity NEXT of NEXT daily NEXT life) OR (daily NEXT living NEXT activit*) OR (daily NEXT life NEXT activit*) OR adl OR (chronic NEXT limitation NEXT of NEXT activity) OR (self NEXT care*) OR (health NEXT status) OR (level NEXT of NEXT health) OR (health NEXT level*) OR qol OR hrql OR hrqol) AND (oral)) OR OHRQoL):ab,ti,kw** | 15,358 |
| #3 | **((oral NEXT function*) OR (oral NEXT dyskinesia*) OR (orofacial NEXT function*) OR (orofacial NEXT dyskinesia*) OR mastication OR chewing OR (tooth NEXT mobilit*) OR (jaw NEXT mobilit*) OR (mandibular NEXT mobilit*) OR (tooth NEXT movement*) OR (jaw NEXT movement*) OR (mandibular NEXT movement*) OR (orofacial NEXT pain) OR (craniofacial NEXT pain) OR (myofacial NEXT pain) OR (facial NEXT pain) OR (neuropathic NEXT pain) OR (burning NEXT mouth) OR (craniomandibular NEXT disorder*) OR (cranio-mandibular NEXT disorder*) OR neuralgia OR trigeminal OR (Tic NEXT Douloureux) OR ("temporomandibular joint" NEXT dis*) OR ("temporo-mandibular joint" NEXT dis*) OR (temporomandibular NEXT dysfunction*) OR (temporo-mandibular NEXT dysfunction*) OR (temporomandibular NEXT disorder*) OR (temporo-mandibular NEXT disorder*) OR (TMJ NEXT dis*) OR TMD):ab,ti,kw** | 12,875 |
| #2 | **((oral NEXT health) OR (oral NEXT hygiene) OR dental OR dentistry OR mouth OR tooth OR teeth OR jaw OR jaws OR periodont* OR parodont* OR (Pyorrhea NEXT Alveolaris) OR periapical OR gingiva* OR gingivi* OR ((gum OR gums) NEAR/3 (inflammat* OR disease*)) OR caries OR carious OR edentulous OR prosthes* OR prosthetic* OR prosthodont*):ab,ti,kw** | 77,022 |
| #1 | **parkinson*:ab,ti,kw** | 11,806 |
